# Signes respiratoires révélant un abcès hépatique amibien

**DOI:** 10.11604/pamj.2015.21.67.7028

**Published:** 2015-05-28

**Authors:** Mohamed Mattous, Khalil Mounir

**Affiliations:** 1 Service des Urgences du 4^ème^ Hôpital Militaire de Dakhla, Dakhla, Maroc; 2 Service de Réanimation du 4^ème^ Hôpital Militaire de Dakhla, Dakhla, Maroc

**Keywords:** Abcès hépatique, signes respiratoires, scanner, liver abscess, Respiratory signs, scanner

## Image en medicine

L'abcès hépatique amibien se définit comme une collection suppurée située au sein du parenchyme hépatique, il est secondaire à l'amibiase colique par Entamoeba histolytica histolytica dont la dissémination se fait par la circulation portale. Il peut être révélé par des signes respiratoires. Son diagnostic repose sur l'imagerie et la sérologie. Le traitement est médical à l'aide du métronidazole, l’évacuation de l'abcès peut être chirurgicale ou percutanée guidée par échographie ou par tomodensitométrie. Nous rapportons un cas d'un patient âgé de 44 ans, ayant présenté il y'a 45 jours une diarrhée mal traitée, admis aux urgences pour dyspnée, toux et fièvre évoluant depuis 7 jours. Le bilan initial avait conclu à un sépsis à point de départ pulmonaire devant la présence d'un syndrome infectieux clinique, une hyperleucocytose à 19500/mm3, une CRP à 183mg/l, et une image en faveur d'un foyer pulmonaire basal bilatéral. Une antibiothérapie par ceftriaxone 2g/24h a été administrée, mais devant l'aggravation de tableau clinique une TDM abdominale a été réalisée et a mis en évidence une masse hépatique du segment IV de densité liquidienne, bien limitée, non rehaussée après injection du produit de contraste iodé, cette masse renferme des bulles aériennes permettant d’évoquer le diagnostic d'abcès hépatique. Devant ce tableau, le métronidazole 500 mg/08h a été associé et le drainage chirurgical a été réalisé ramenant un liquide brun chocolat dont l’étude parasitologique était négative. La sérologie pour Entamoeba histolytica histolytica était positive. Le patient est décédé suite à une hypoxie réfractaire sur syndrome de détresse respiratoire aigue.

**Figure 1:**
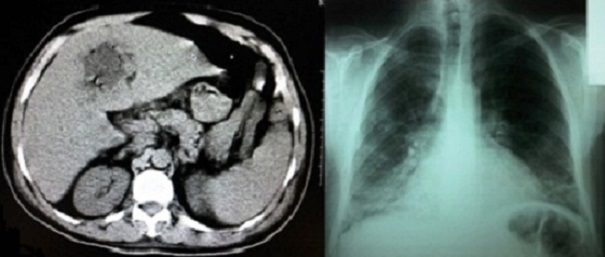
Tomodensitométrie abdominale avec injection de produit de contraste iodé, coupe axiale, montrant une masse hépatique de segment IV, radiographie pulmonaire de face montrant une image en faveur d'un foyer pulmonaire basal bilatéral

